# Score-based prediction model for severe vitamin D deficiency in patients with critical illness: development and validation

**DOI:** 10.1186/s13054-022-04274-9

**Published:** 2022-12-21

**Authors:** Yu-Ting Kuo, Li-Kuo Kuo, Chung-Wei Chen, Kuo-Ching Yuan, Chun-Hsien Fu, Ching-Tang Chiu, Yu-Chang Yeh, Jen-Hao Liu, Ming-Chieh Shih

**Affiliations:** 1 Department of Anesthesiology, National Taiwan University Hospital, No. 7, Zhongshan S. Rd., Taipei City, 10002 Taiwan; 2 Division of Critical Care Medicine, Mackay Memorial Hospital, No. 92, Sec. 2, Zhongshan N. Rd., Taipei City, Taiwan; 3 Department of Medicine, Mackay Medical College, No. 46, Sec. 3, Zhongzheng Rd., Sanzhi Dist., New Taipei City, Taiwan; 4 Department of Surgical Intensive Care Unit, Far Eastern Memorial Hospital, No. 21, Sec. 2, Nanya S. Rd., Banciao Dist., New Taipei City, Taiwan; 5 Department of Critical Care Medicine, Taipei Medical University Hospital, No. 252, Wuxing St, Taipei City, Taiwan; 6 Department of Anesthesiology, Fu Jen Catholic University Hospital, No. 69, Guizi Road, New Taipei City, Taiwan; 7 Department of Applied Mathematics, College of Science and Engineering, National Dong Hwa University, No. 1-12, Sec. 2, University Rd., Hualien County, 974 Taiwan

**Keywords:** Vitamin D, Critical care, Deficiency, Prediction model

## Abstract

**Background:**

Severe vitamin D deficiency (SVDD) dramatically increases the risks of mortality, infections, and many other diseases. Studies have reported higher prevalence of vitamin D deficiency in patients with critical illness than general population. This multicenter retrospective cohort study develops and validates a score-based model for predicting SVDD in patients with critical illness.

**Methods:**

A total of 662 patients with critical illness were enrolled between October 2017 and July 2020. SVDD was defined as a serum 25(OH)D level of < 12 ng/mL (or 30 nmol/L). The data were divided into a derivation cohort and a validation cohort on the basis of date of enrollment. Multivariable logistic regression (MLR) was performed on the derivation cohort to generate a predictive model for SVDD. Additionally, a score-based calculator (the SVDD score) was designed on the basis of the MLR model. The model’s performance and calibration were tested using the validation cohort.

**Results:**

The prevalence of SVDD was 16.3% and 21.7% in the derivation and validation cohorts, respectively. The MLR model consisted of eight predictors that were then included in the SVDD score. The SVDD score had an area under the receiver operating characteristic curve of 0.848 [95% confidence interval (CI) 0.781–0.914] and an area under the precision recall curve of 0.619 (95% CI 0.577–0.669) in the validation cohort.

**Conclusions:**

This study developed a simple score-based model for predicting SVDD in patients with critical illness.

*Trial registration*: ClinicalTrials.gov protocol registration ID: NCT03639584. Date of registration: May 12, 2022.

**Graphical Abstract:**

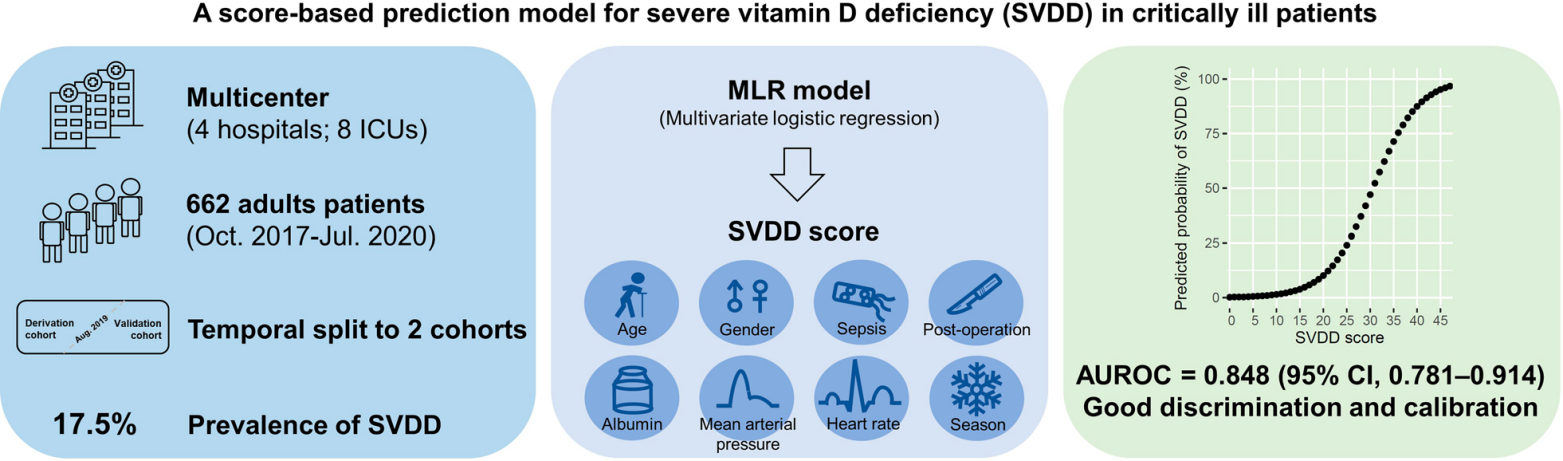

**Supplementary Information:**

The online version contains supplementary material available at 10.1186/s13054-022-04274-9.

## Introduction

Severe vitamin D deficiency (SVDD), defined as a 25-hydroxy-vitamin D [25(OH)D] concentration below 12 ng/mL (or 30 nmol/L), is highly prevalent in patients admitted to intensive care units (ICUs) and is associated with adverse outcomes [1–3]. The prevalence of SVDD in ICUs typically ranges from 20 to 70% [4]. In Taiwan, the prevalence of vitamin D deficiency [i.e., 25(OH)D level below 20 ng/mL or 50 nmol/mL] in the general population ranges from 20 to 40% [5–7]; however, there are little data about the prevalence of SVDD. Additionally, our previous multicenter observational study reported a higher prevalence of vitamin D deficiency of 59% and a prevalence of SVDD of 18% in critically ill patients in Taiwan [8]. The study also revealed strong associations of vitamin D deficiency with longer duration of ventilator use and greater length of ICU stay [8].

Supplementation of vitamin D in patients with critical illness has been reported to be safe [9]. According to the 2019 European Society for Clinical Nutrition and Metabolism guidelines for clinical nutrition in the ICU [10], administering a single high dose of vitamin D3 (500,000 UI) in patients with vitamin D deficiency is recommended within 1 week of ICU admission. However, vitamin D testing for every ICU patient is not a routine practice and may be impractical and too expensive in many countries. Therefore, developing a prediction model for SVDD to determine which patient would benefit most from vitamin D tests and supplementation is essential.

Several models for predicting vitamin D deficiency have been created for the general population [11–14] but not patients admitted to ICUs. To facilitate decision making on vitamin D supplementation in an intensive care setting, this multicenter cohort study developed and validated a score-based model for predicting SVDD in patients with critical illness.

## Methods

### Study design

This study was based on the data obtained in our previous multicenter, prospective, observational study [8]. It was approved by the Research Ethics Committee of National Taiwan University Hospital (approval number: 202203073RIND) and registered on the ClinicalTrials.gov protocol registration system (ID: NCT05376774). This study was conducted in eight ICUs at four hospitals in northern Taiwan between October 2017 and July 2020. We included surgical ICUs (SICUs), medical ICUs (MICUs), and mixed ICUs with both postoperative patients and medical cases. To perform temporal validation, the data were divided into a derivation cohort (the first 77% of the data set) and a validation cohort (the remaining 23% of the data set) on the basis of the date of enrollment. To cover all seasons, the validation cohort included patients over a year (i.e., August 2019 to July 2020). The models were developed and validated in accordance with the recommendations established in the Transparency Reporting of a multivariable prediction model for Individual Prognosis or Diagnosis (TRIPOD) initiative [15].

### Study sample

The inclusion and exclusion criteria were the same as in our previous study [8]. Patients admitted to the ICUs were eligible for enrollment. Patients were excluded if they were younger than 20 years old; had a body mass index lower than 18 kg/m^2^; had severe anemia (i.e., a hemoglobin level less than 7 g/dL); received an additional vitamin D supplement greater than 3,000 IU/day within 4 weeks of the study; were previously admitted to an ICU within the preceding 3 months; or had a diagnosis of hyperparathyroidism, rickets, or liver cirrhosis (Child–Pugh C). Because the study was a retrospective analysis of deidentified collected data, informed consent was not required.

### Predictor selection and processing

We selected 15 variables as candidate predictors on the basis of our clinical judgment and a literature review [11–13]. To facilitate practical application of the models, we excluded variables that are not routinely recorded or tested, such as C-reactive protein and total serum calcium levels. Imbalanced categorical predictors with percentages smaller than 10% or greater than 90% were excluded to prevent overfitting [16]. Ultimately, six categories of candidate predictors were incorporated into our models: general characteristics, comorbidities, indication of ICU admission, enrollment season, vital signs, and laboratory findings. All the data were collected upon enrollment. Multiple imputation was conducted using the “mice” package to address missing data for the potential predictors [17]. Five imputed data sets were created; the imputation methods consisted of predictive mean matching for continuous predictors and logistic regression for binary predictors. All statistical analyses were performed using the five imputed data sets, and the estimates were combined in accordance with the guidelines proposed by Marshall et al. [18].

### Outcome definition

The outcome variable, SVDD, was defined as a serum 25(OH)D level of < 12 ng/mL (or 30 nmol/L). In our previous study, blood samples were obtained upon enrollment, and serum 25(OH)D level was measured using the commercially available TOTAL Liaison chemiluminescence assay (Liaison, Diasorin, Saluggia, Italy) [19].

### Statistical analysis

We express categorical variables as percentages and continuous variables as medians. The Shapiro–Wilk test was used to test normality. For comparisons between the derivation and validation cohorts, we performed the Mann–Whitney test for non-normally distributed continuous variables, Student’s *t* test for normally distributed continuous variables, and the Chi-squared test for categorical variables. A *P* value of < 0.05 was considered statistically significant.

We first fit a multivariable logistic regression (MLR) model in the derivation cohort. The relationships between heart rate, age, and outcome could be nonlinear; this was enabled by the use of restricted cubic splines [20]. To construct a simple prediction score (i.e., the SVDD score), we included predictors that were significantly associated with SVDD in the MLR model [21–23] or predictors that strongly influenced the model, that is, that had a standardized odds ratio greater than 1.2 or less than 0.8. A reduced MLR model was created using these included predictors, and SVDD scores were estimated using the reduced MLR model.

To convert the SVDD scores into integers, the regression *β* coefficients were multiplied by 5 and rounded to the nearest integer [22, 23]. We wanted a score of 0 for the lowest-risk group. By grouping each continuous predictor into convenient intervals, such as intervals of 10 mmHg for mean blood pressure, an individual's score increased by an integer amount for each risk factor level above the lowest-risk category [21]. The total number of points was the value of the final SVDD score. Additionally, estimated probabilities of each SVDD score were obtained using logistic regression.

The performance of the MLR model and the SVDD score were evaluated in both the derivation and validation cohorts by using metrics that represent discrimination and calibration. Discrimination was assessed using the area under the receiver operating characteristic curve (AUROC) and the area under the precision recall curve (AUPRC). Calibration was assessed using calibration plots, Brier scores, and Hosmer and Lemeshow goodness-of-fit tests. Post hoc recalibrations were performed by adjusting the intercept because the predicted probability was underestimated, which resulted from differences in the overall incidence of SVDD between the derivation and validation cohorts [15, 24].

Patients were divided into three risk groups: very low risk, low risk, and medium-to-high risk groups. The groups were based on the estimated probabilities [25]. We also developed web and mobile phone applications with which clinicians can calculate SVDD scores and conveniently interpret the risk stratification. All statistical analyses were conducted using R (R version 4.1.3; R Foundation for Statistical Computing, Vienna, Austria).

## Results

### Patient enrollment and characteristics

**Fig. 1:**
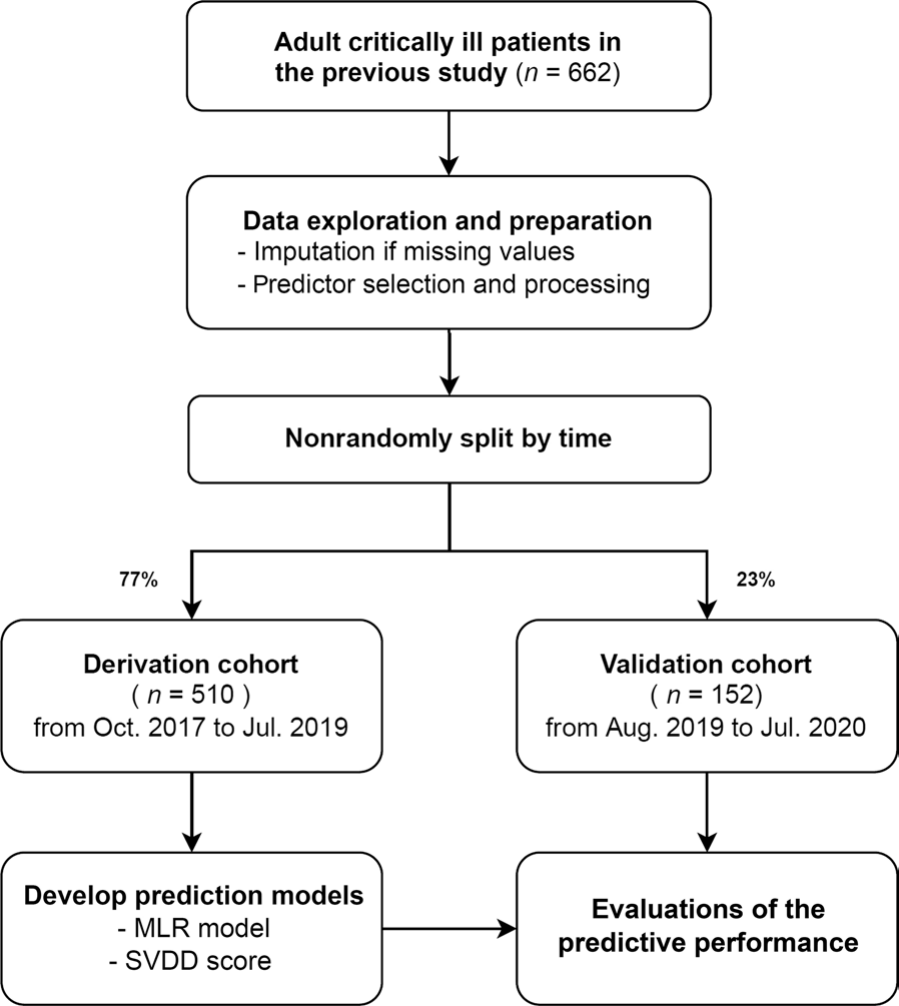
Scheme of the development and validation of the prediction model. Abbreviations: MLR, multivariable logistic regression; SVDD, severe vitamin D deficiency

We divided 662 patients from our previous study into a derivation cohort with 510 patients (from October 2017 to July 2019) and a validation cohort with 152 patients (from August 2019 to July 2020; Fig. 1). Table 1 shows the patient characteristics of both cohorts. The significant differences found in the validation cohort compared with the derivation cohort were a lower percentage of neurological indication of ICU admission (3.3% vs. 11.3%), a lower percentage of enrollment in spring (13.2% vs. 28.6%), a higher percentage of enrollment in fall (30.9% vs. 20.4%), a lower median total calcium level (2.07 vs. 2.10 mmol/L), and a higher median C-reactive protein level (11.7 vs. 6.7 mg/L). The prevalence of SVDD was 16.3% and 21.7% in the derivation and validation cohorts (*P* = 0.154), respectively.

**Table 1:** Baseline characteristics and risk factors in derivation and validation cohorts

Characteristics	Missing value, *n*	All patients (*n* = 662)	Derivation cohort (*n* = 510)	Validation cohort (*n* = 152)	*P* value
General characteristics					
Age (years)	0 (0%)	67 (56–78)	67 (56–78)	68 (56–78)	0.558^a^
Men	0 (0%)	405 (61.2%)	319 (62.5%)	86 (56.6%)	0.218^b^
BMI (kg/m^2^)	1 (0.2%)	24 (21–27)	24 (21–27)	24 (21–27)	0.808^a^
Comorbidities					
ESRD	0 (0%)	37 (5.6%)	27 (5.3%)	10 (6.6%)	0.686^b^
Diabetes	0 (0%)	202 (30.5%)	154 (30.2%)	48 (31.6%)	0.822^b^
Cerebrovascular disease	0 (0%)	90 (13.6%)	76 (14.9%)	14 (9.2%)	0.096^b^
Liver disease	0 (0%)	35 (5.3%)	26 (5.1%)	9 (5.9%)	0.848^b^
Metastasis	0 (0%)	48 (7.3%)	34 (6.7%)	11 (9.2%)	0.377^b^
Indication of ICU admission					
Sepsis	1 (0.2%)	117 (17.7%)	87 (17.1%)	30 (19.7%)	0.523^b^
Postoperation	1 (0.2%)	330 (49.8%)	246 (48.2%)	84 (55.3%)	0.153^b^
Respiratory	1 (0.2%)	54 (8.2%)	39 (7.6%)	15 (9.9%)	0.478^b^
Cardiovascular	1 (0.2%)	33 (5.0%)	28 (5.5%)	5 (3.3%)	0.378^b^
Neurological	1 (0.2%)	63 (9.5%)	58 (11.3%)	5 (3.3%)	0.005^b^
Enrollment season					< 0.001^b^
Spring (March to May)	0 (0%)	166 (25.1%)	146 (28.6%)	20 (13.2%)	
Summer (June to August)	0 (0%)	181 (27.3%)	136 (26.7%)	45 (29.6%)	
Fall (September to November)	0 (0%)	151 (22.8%)	104 (20.4%)	47 (30.9%)	
Winter (December to February)	0 (0%)	164 (24.8%)	124 (24.3%)	40 (26.3%)	
Vital signs					
MAP (mmHg)	0 (0%)	88 (78–97)	88 (79–97)	87 (76–96)	0.305^c^
Heart rate (/min)	0 (0%)	88 (76–100)	87 (76–100)	89 (77–101)	0.300^a^
Laboratory finding					
Lactate (mmol/L)	133 (20.1%)	1.9 (1.2–7.5)	1.9 (1.2–7.6)	1.8 (1.3–6.0)	0.603^a^
Albumin (g/dL)	107 (16.2%)	3.1 (2.7–3.5)	3.1 (2.7–3.5)	3.0 (2.7–3.5)	0.242^a^
WBC (k/μL)	0 (0%)	10.6 (8.1–14.3)	10.6 (8.1–14.1)	11.1 (8.2–14.7)	0.585^a^
Hemoglobin (g/dL)	0 (0%)	10.2 (9.1–11.5)	10.2 (9.1–11.6)	9.9 (8.9–11.2)	0.077^a^
Platelet (k/μL)	0 (0%)	178 (122–252)	184 (126–257)	160 (120–227)	0.075^a^
Sodium (mmol/L)	0 (0%)	138 (135–141)	138 (135–141)	137 (134–140)	0.081^a^
Potassium (mmol/L)	0 (0%)	3.9 (3.5–4.2)	3.9 (3.5–4.2)	3.8 (3.4–4.1)	0.140^a^
Total calcium (mmol/L)	94 (14.2%)	2.1 (2.0–2.2)	2.1 (2.0–2.2)	2.1 (2.0–2.2)	0.007^a^
Creatinine (mg/dL)	0 (0%)	1.0 (0.7–1.7)	1.0 (0.7–1.7)	0.9 (0.7–1.7)	0.874^a^
CRP (mg/L)	276 (41.7%)	6.9 (2.4–16.8)	6.7 (2.1–15.9)	11.7 (3.8–19.4)	0.013^a^
Vitamin D (ng/mL)	0 (0%)	18.3 (13.7–23.9)	18.7 (13.9–24.0)	17.5 (12.5–23.6)	0.232^a^
SVDD	0 (0%)	116 (17.5%)	83 (16.3%)	33 (21.7%)	0.154^b^

*BMI* body mass index, *ESRD* end stage renal disease, *MAP* mean arterial pressure, *WBC* white blood cell, *CRP* C-reactive protein, *SVDD* severe vitamin D deficiency

Data are presented as number (%), or median (interquartile range). Statistical methods: ^a^Mann–Whitney test; ^b^Chi-squared test; ^c^Student’s *t* test

### MLR analyses and development of prediction models

Additional file 1: Table S1 summarizes the result of the MLR analysis. Restricted cubic splines were applied to age and heart rate. The spline variables were prepared using four knots set at the 5th, 35th, 65th, and 95th percentiles of the variables. Significant contributions were made by age, gender, heart rate, sepsis, albumin level, and mean arterial pressure. Additionally, postoperation, that is entering the ICU after having received surgery, and enrollment season greatly influenced the model. These eight predictors and their *β* coefficients in the reduced MLR model were then used to determine the SVDD scores. Table 2 presents the score chart. The SVDD score was defined as the sum of the points from each variable.

**Table 2:** SVDD score chart

	Addition to the SVDD score													
Age (years)	< 55 ** + 7**	55–59 ** + 6**	60–64 ** + 4**	65–69 ** + 2**	70–74 ** + 1**	75–79 ** + 0**	80–84 ** + 1**	85–89 ** + 2**	90–94 ** + 3**	≥ 95 ** + 4**				
MAP (mmHg)	< 60 ** + 0**	60–69 ** + 1**	70–79 ** + 2**	80–89 ** + 3**	90–99 ** + 4**	100–109 ** + 5**	110–119 ** + 6**	120–129 ** + 7**	≥ 130 ** + 8**					
Albumin (g/dL)	< 1.5 ** + 10**	1.5–1.9 ** + 9**	2.0–2.4 ** + 8**	2.5–2.9 ** + 6**	3.0–3.4 ** + 5**	3.5–3.9 ** + 3**	4.0–4.4 ** + 2**	≥ 4.5 ** + 0**						
HR (/min)	< 75 ** + 4**	75–79 ** + 5**	80–84 ** + 6**	85–89 ** + 8**	90–94 ** + 9**	95–99 ** + 10**	100–109 ** + 9**	110–114 ** + 8**	115–119 ** + 6**	120–124 ** + 5**	125–129 ** + 4**	130–134 ** + 3**	135–139 ** + 1**	≥ 140 ** + 0**
Gender	Male	** + 0**	Female	** + 4**										
Sepsis	No	** + 0**	Yes	** + 4**										
Postoperation	No	** + 1**	Yes	** + 0**										
Season	Spring	** + 1**	Summer	** + 0**	Fall	** + 3**	Winter	** + 1**						

*MAP* mean arterial pressure, *HR* heart rate, *SVDD* severe vitamin D deficiency. Values in bold indicate extra points added to the SVDD score

### Performance analyses

SVDD score had an AUROC of 0.751 [95% confidence interval (CI) 0.694–0.809] in the derivation cohort and 0.848 (95% CI 0.781–0.914) in the validation cohort, neither of which were significantly different from the AUROC of the MLR models. The AUPRC of SVDD score was 0.439 (95% CI 0.381–0.491) in the derivation cohort and 0.619 (95% CI 0.577–0.669) in the validation cohort. The calibration plots for the MLR model and SVDD score presented in Additional file 1: Figs. S1 and S2 indicated acceptable calibration in the derivation cohort. However, the calibration plots for SVDD score in the validation cohort revealed a general trend of underestimation and required recalibration with the intercept. We obtained the recalibrated intercept of 0.273 (*P* = 0.203). The performance of the recalibrated model is illustrated in Additional file 1: Fig. S2.

### Application of SVDD score prediction model

**Fig. 2:**
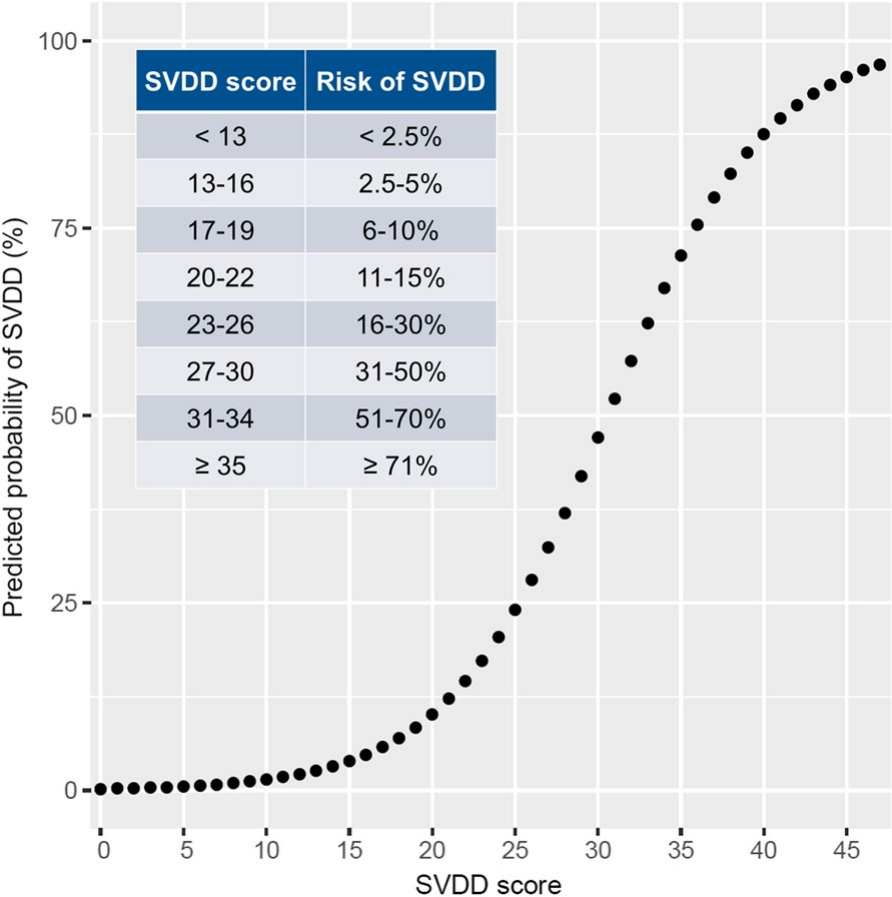
Recalibrated predicted probabilities of SVDD for each SVDD score. Abbreviations: SVDD, severe vitamin D deficiency

Figure 2 shows the SVDD score predictions. The estimated probability of SVDD was calculated as follows:

$$\frac{1}{{1 + e^{{ - \left[ { - 6.58 + 0.21 \times SVDD\;score} \right]}} }}.$$

The recalibrated probability of SVDD was calculated as follows:

$$\frac{1}{{1 + e^{{ - \left[ {0.273 - 6.58 + 0.21 \times SVDD\;score} \right]}} }}.$$

**Fig. 3:**
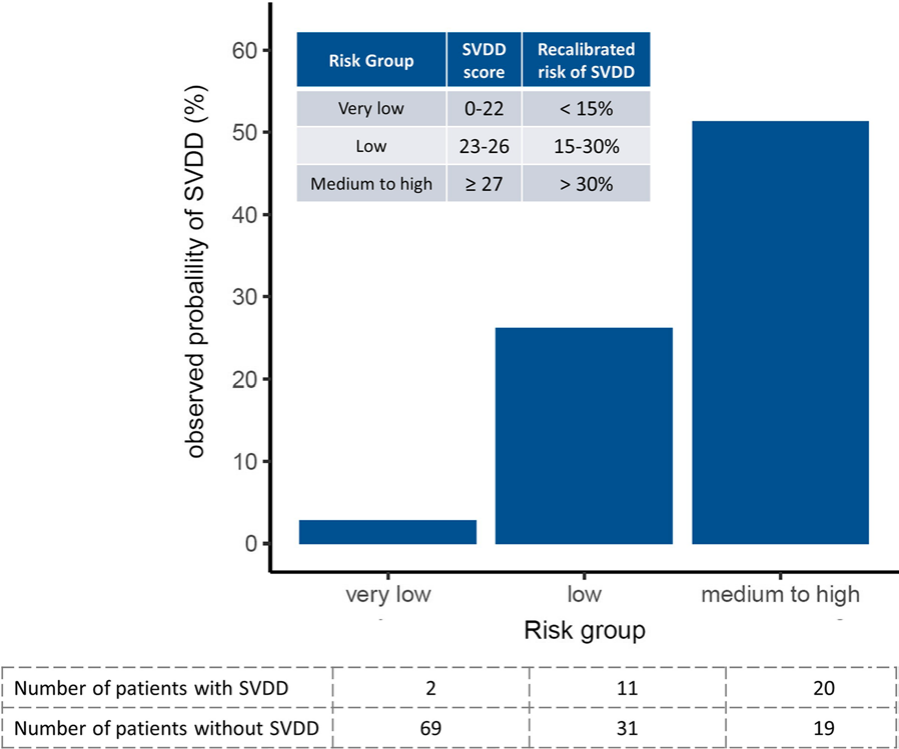
Probability of SVDD in accordance with risk groups in the validation cohort. Abbreviations: SVDD, severe vitamin D deficiency

The recalibrated probability of SVDD was grouped into three categories: very low risk (≤ 15%), with an SVDD score of 0–22; low risk (15–30%), with an SVDD score of 23–26; and medium-to-high risk (≥ 30%), with an SVDD score of ≥ 27 (Fig. 3). Applying the risk group classification to the validation cohort revealed favorable discrimination and an AUROC of 0.812 (95% CI 0.741–0.883). When using a score of 24 as the threshold for predicting SVDD, the sensitivity was 86% (95% CI 70–96%), the specificity was 65% (95% CI 55–73%), the positive predictive value was 40% (95% CI 28–41%), and the negative predictive value was 94% (95% CI 90–98%). Additional file 1: Figs. S3 and S4 show the web [26] and mobile phone applications for the SVDD scores.

## Discussion

This multicenter cohort study constructed a score-based model for predicting SVDD in patients with critical illness. Independent predictors of SVDD include age, gender, sepsis, postoperation, season, heart rate, mean arterial pressure, and albumin level. The SVDD score demonstrated favorable performance, with its AUROC being 0.848 and exhibited good calibration after recalibration. Our model can predict SVDD in patients with critical illness by calculating a simple SVDD score and can assist with screening high-risk patients who may benefit from vitamin D supplementation [27].

The SVDD score is an easy-to-use scoring tool and is based on information routinely available in ICUs. A patient’s risk of SVDD is quantified by simply using an SVDD scoring chart (Table 2) and the predicted probability of each SVDD score (Fig. 2); complex computer calculations are not required. We also developed web and mobile phone application that had an SVDD score calculator; clinicians can use the web or application to conveniently assess a patient’s risk of SVDD. Clearly defined risk groups were established and demonstrated to have favorable discrimination ability. In some countries, vitamin D level tests are time-consuming and expensive; the proposed SVDD score can facilitate vitamin D supplementation for patients with critical illness and reduce the money spent on vitamin D tests. It has wide applicability in general ICU practice. Kheir et al. had proposed a single-center study about a dynamic nomogram predicting SVDD at ICU admission [28]. In comparison with our SVDD score, their model depended on complex computer calculation, and the predictors included other clinical scores that needs further calculation, such as Sequential Organ Failure Assessment score.

Considering the population of patients with critical illness and the feasibility of use of the prediction model in ICUs, we excluded some predictors that are commonly included in vitamin D deficiency models, such as suntan use, fatty fish consumption, or lifestyle. Body mass index was a potential predictor but found to not be significantly associated with SVDD. Our results revealed that female gender, sepsis, hypoalbuminemia, and high mean arterial pressure are significantly associated with SVDD. These findings are consistent with those of other studies [29–32]. Moreover, age and heart rate had nonlinear relationships with SVDD in our prediction models. In other studies [11–14], age has often been dichotomized using variable cutoffs, although the TRIPOD guidelines strongly discourage the dichotomization of continuous predictors [15]. Further studies are necessary to investigate the mechanism or possible confounding effects of this nonlinear relationship. In our study, postoperation was a protective predictor for SVDD. We suggest that medical cases have more comorbidities than postoperative patients, and multimorbidity may be a risk factor of vitamin D deficiency. Further studies are warranted to investigate SVDD in patients admitted to SICU or MICU.

The strengths of this study include a multicenter design, the use of predictors that can feasibly be determined in an ICU setting, and strict adherence to the TRIPOD guidelines. The limitations of this study are a small sample, few events per predictor in the MLR model, missing values for some of the laboratory data, and heterogeneity of patients from different types of ICUs. Moreover, the prediction model lacks external validity, and the model may not be applicable in countries at different latitudes or in specialized ICUs. Recalibrations may be required for new study populations and settings. Future studies are warranted to externally validate the SVDD prediction model.

## Conclusions

Our study establishes an easy-to-use SVDD score for predicting SVDD in patients admitted to ICUs. This SVDD score is the first vitamin D deficiency prediction score that is specialized to patients with critical illness. Future studies in different countries and geographic locations are necessary to externally validate the model.

## Supplementary Information

**Additional file 1.** Results of multivariable logistic regression analyses, model performance, the score calculator website, and phone application.

## Data Availability

The data used during the current study are available from the corresponding author on reasonable request after obtaining the agreement of Research Ethic Committee of National Taiwan University Hospital. All codes and processes used in the current study are available from the corresponding author on reasonable request.

## Abbreviations

SVDD: Severe vitamin D deficiency
MLR: Multivariable logistic regression
AUROC: Area under the receiver operating characteristic curve
CI: Confidence interval
AUPRC: Area under precision recall curve
ICU: Intensive care unit
SICU: Surgical intensive care unit
MICU: Medical intensive care unit
ESPEN: European Society for Clinical Nutrition and Metabolism
TRIPOD: Transparency Reporting of a multivariable prediction model for Individual Prognosis or Diagnosis
BMI: Body mass index
CRP: C-reactive protein
ESRD: End stage renal disease
MAP: Mean arterial pressure
HR: Heart rate
WBC: White blood cell
GOF: Goodness-of-fit
