# Supplementary material for: Score-based prediction model for severe vitamin D deficiency in patients with critical illness: development and validation
Source: Crit Care. 2022 Dec 21;26:394. doi: 10.1186/s13054-022-04274-9 (PMC9768894; doi:10.1186/s13054-022-04274-9)
Supplement: Supplementary file 1 — Additional file 1. Results of multivariable logistic regression analyses, model performance, the score calculator website, and phone application. [file 13054_2022_4274_MOESM1_ESM.docx]

**Additional file 1**

Supplement to:

**Score-based prediction model for severe vitamin D deficiency in patients with critical illness: Development and validation**

Yu-Ting Kuo, Li-Kuo Kuo, Chung-Wei Chen, Kuo-Ching Yuan, Chun-Hsien Fu, Ching-Tang Chiu, Yu-Chang Yeh, Jen-Hao Liu, Ming-Chieh Shih

Corresponding authors (equal contribution):

Dr. **Jen-Hao Liu**

Department of Anaesthesiology, National Taiwan University Hospital

No 7, Zhongshan S. Rd, Taipei, Taiwan

ORCID: 0000-0002-6421-9568

E-mail: b98401003@gmail.com

Dr. **Ming-Chieh Shih**

Institute of epidemiology and preventive medicine college of public health, National Taiwan University

No.17, Xu-Zhou Rd., Taipei City 100, Taiwan

E-mail: littlecanargie@gmail.com

**Content**

**1. Supplementary Tables**

Table S1 Multivariable logistic regression analyses for predicting SVDD

**2. Supplementary Figures**

Figure S1 Model performance and calibration of reduced multivariate logistic regression model

Figure S2 Model performance and calibration of the SVDD score

Figure S3 Display of SVDD score calculator website

Figure S4 Display of the SVDD score calculator phone application

**Table S1 Multivariable logistic regression analyses for predicting SVDD**

|  | Full MLR model (15 predictors) | | | | Reduced MLR model (8 predictors) | | |
| --- | --- | --- | --- | --- | --- | --- | --- |
| Intercept and predictors | *β* | Odds ratio  (95% CI) | Odds ratio (95% CI) with continuous predictors standardized | *P* value | *β* | Odds ratio  (95% CI) | *P* value |
| Intercept | 7.324 | - | - | - | -1.013 | - | - |
| Age (spline 1)* | -0.001 | - | - | <0.001 | 0.005 | - | 0.001 |
| Age (spline 2)* | -0.101 | - | - |  | -0.100 | - |  |
| Age (spline 3)* | 0.774 | - | - |  | 0.741 | - |  |
| Men | -0.776 | 0.46 (0.27-0.79) | 0.47 (0.27-0.80) | 0.005 | -0.803 | 0.45 (0.26-0.76) | 0.003 |
| BMI | -0.030 | 0.97 (0.92-1.02) | 0.87 (0.66-1.13) | 0.281 | - | - | - |
| Sepsis | 0.766 | 2.15 (1.03-4.52) | 2.09 (1.00-4.38) | 0.043 | 0.767 | 2.15 (1.09-4.26) | 0.028 |
| Post-operation | -0.269 | 0.76 (0.42-1.38) | 0.77 (0.43-1.40) | 0.374 | -0.273 | 0.76 (0.43-1.26) | 0.357 |
| Season (ref=summer) |  |  |  |  |  |  |  |
| Spring | 0.239 | 1.27 (0.60-2.68) | 1.26 (0.60-2.67) | 0.531 | 0.130 | 1.14 (0.56-2.33) | 0.722 |
| Fall | 0.554 | 1.74 (0.79-3.83) | 1.74 (0.79-3.83) | 0.170 | 0.548 | 1.73 (0.80-3.75) | 0.166 |
| Winter | 0.375 | 1.45 (0.69-3.07) | 1.47 (0.70-3.09) | 0.325 | 0.287 | 1.33 (0.64-2.76) | 0.439 |
| MAP | 0.021 | 1.02 (1.00-1.04) | 1.35 (1.03-1.78) | 0.034 | 0.018 | 1.02 (1.00-1.04) | 0.068 |
| Lactate | -0.003 | 1.00 (0.96-1.04) | 1.04 (0.69-1.58) | 0.899 | - | - | - |
| Albumin | -0.513 | 0.60 (0.39-0.91) | 0.73 (0.56-0.96) | 0.018 | -0.603 | 0.55 (0.36-0.82) | 0.004 |
| HR (spline 1)** | -0.008 | - | - | 0.030 | -0.004 | - | 0.030 |
| HR (spline 2)** | 0.143 | - | - |  | 0.129 | - |  |
| HR (spline 3)** | -0.674 | - | - |  | -0.622 | - |  |
| WBC | -0.027 | 0.97 (0.93-1.02) | 0.87 (0.66-1.14) | 0.295 | - | - | - |
| Hemoglobin | -0.115 | 0.89 (0.76-1.05) | 0.81 (0.60-1.10) | 0.158 | - | - | - |
| Platelet | -0.001 | 1.00 (1.00-1.00) | 0.89 (0.68-1.17) | 0.404 | - | - | - |
| Sodium | -0.042 | 0.96 (0.91-1.01) | 0.81 (0.60-1.08) | 0.108 | - | - | - |
| Potassium | -0.023 | 0.98 (0.62-1.55) | 0.98 (0.76-1.28) | 0.923 | - | - | - |

* Knots of the restricted cubic spline of age: 34, 61, 74, 89

** Knots of the restricted cubic spline of heart rate: 61, 81, 94, 114

Abbreviations: CI, confidence interval; BMI, Body Mass Index; MAP, mean arterial pressure; WBC, white blood cell; HR, heart rate

**Figure S1 Model performance and calibration of reduced multivariate logistic regression model**


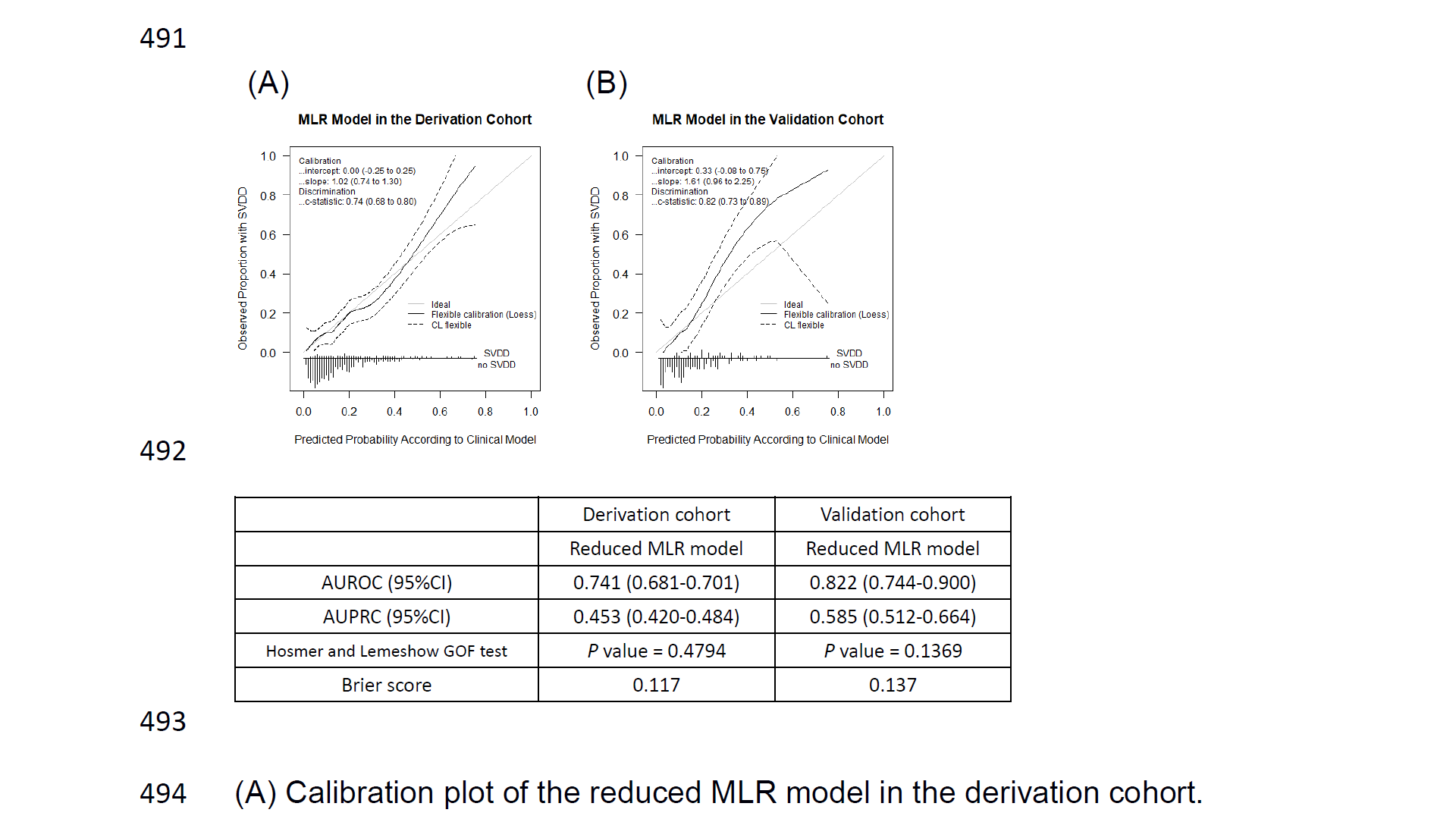


(A) Calibration plot of the reduced MLR model in the derivation cohort.

(B) Calibration plot of the reduced MLR model in the validation cohort.

Abbreviations: MLR, multivariable logistic regression; AUROC, the area under the receiver operating characteristic curve; AUPRC, the area under precision recall curve

**Figure S2 Model performance and calibration of the SVDD score**


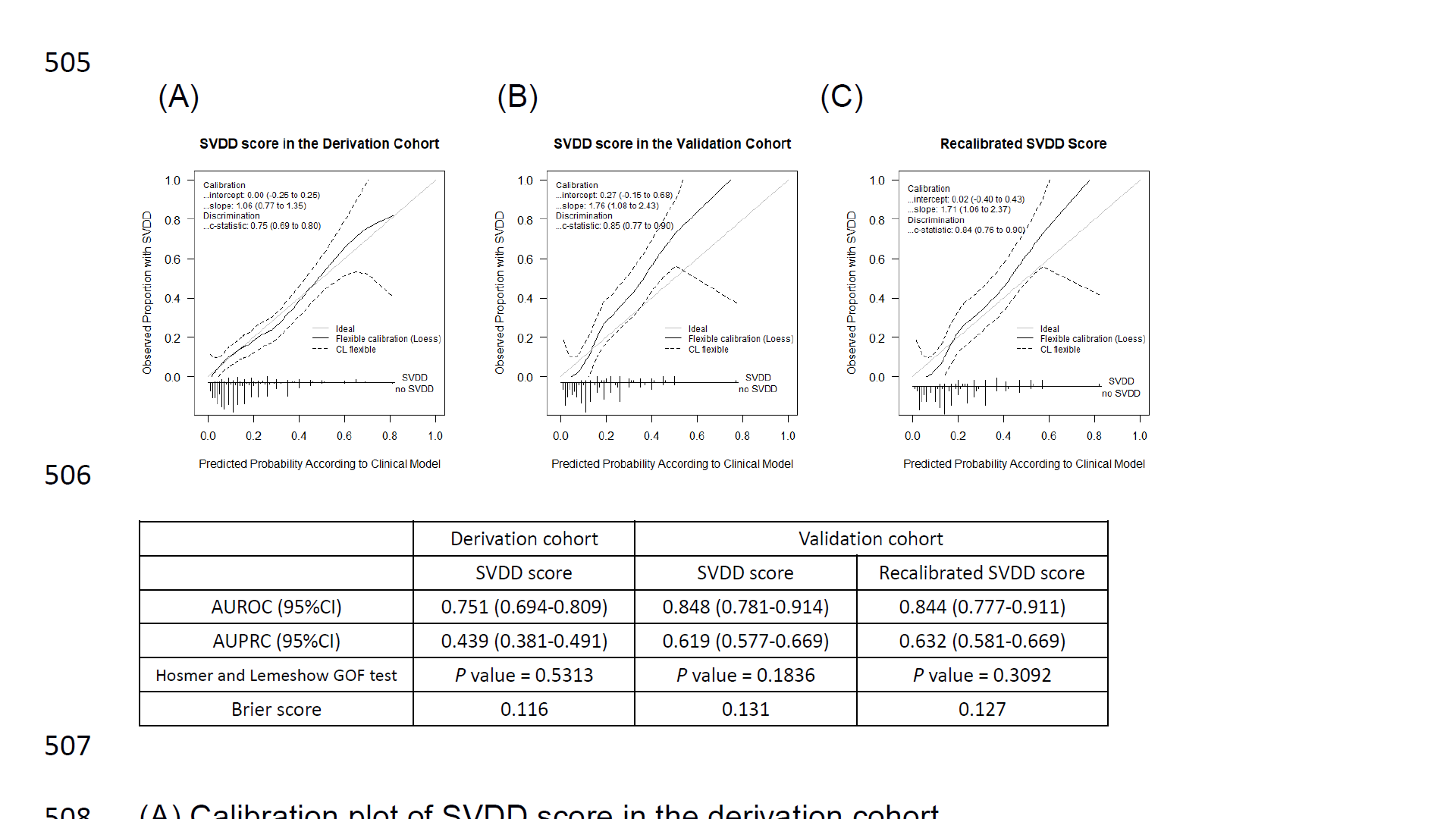


(A) Calibration plot of SVDD score in the derivation cohort.

(B) Calibration plot of the SVDD score in the validation cohort.

(C) Calibration plot of the recalibrated SVDD score in the validation cohort.

Abbreviations: SVDD, severe vitamin D deficiency; AUROC, the area under the receiver operating characteristic curve; AUPRC, the area under precision recall curve.

**Figure S3 Display of SVDD score calculator website**

**
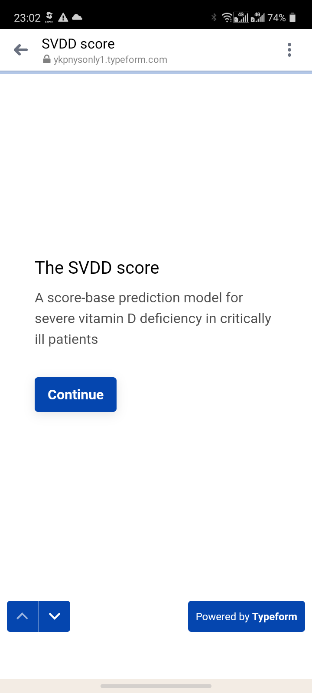

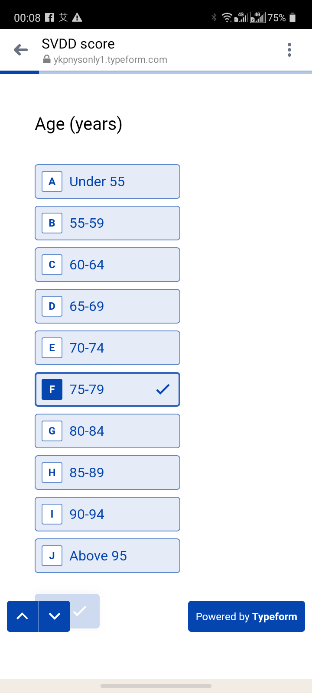

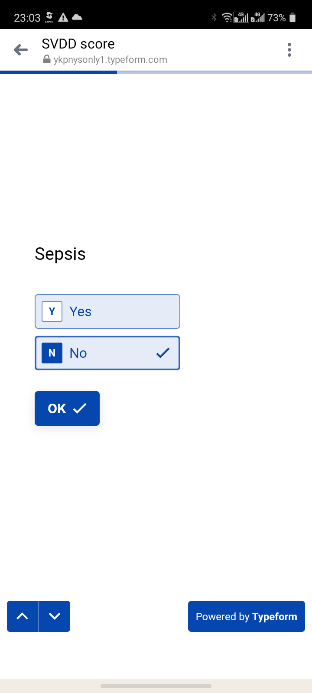

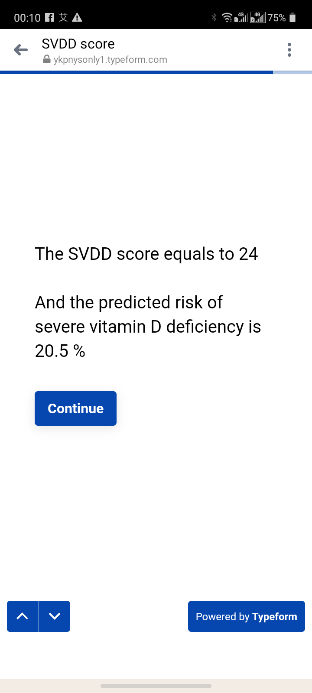
**

The link to our website: <https://shock.icu/SVDD>

**Figure S4 Display of the SVDD score calculator phone application
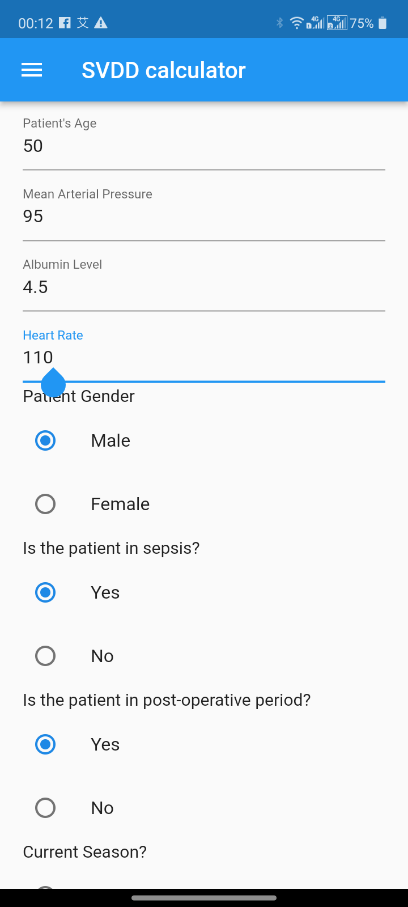

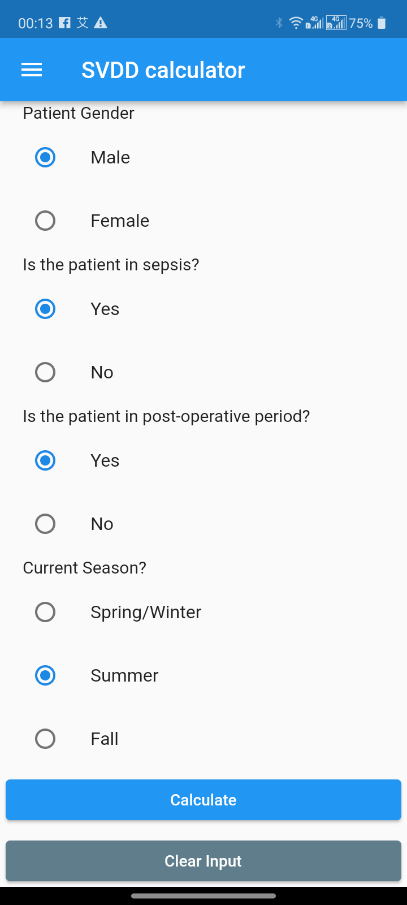

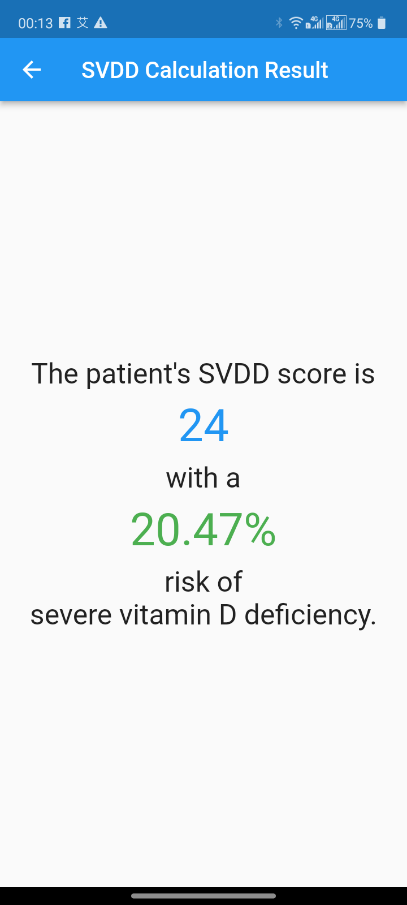
**

The link to download our mobile phone application: <https://ppt.cc/fGwlVx>
